# SensorNet: An Adaptive Attention Convolutional Neural Network for Sensor Feature Learning

**DOI:** 10.3390/s24113274

**Published:** 2024-05-21

**Authors:** Jiaqi Ge, Gaochao Xu, Jianchao Lu, Xu Xu, Long Li, Xiangyu Meng

**Affiliations:** 1 Department of Computer Science and Technology, Jilin University, Changchun 130012, China; gejq18@mails.jlu.edu.cn (J.G.); xugc@jlu.edu.cn (G.X.); xuxu20@mails.jlu.edu.cn (X.X.); lilong21@mails.jlu.edu.cn (L.L.); 2 School of Computing, Macquarie University, Sydney, NSW 2109, Australia; juliuslu2014@gmail.com

**Keywords:** sensor feature learning, human behavior recognition, attention convolutional neural network

## Abstract

This work develops a generalizable neural network, SENSORNET, for sensor feature learning across various applications. The primary challenge addressed is the poor portability of pretrained neural networks to new applications with limited sensor data. To solve this challenge, we design SensorNet, which integrates the flexibility of self-attention with multi-scale feature locality of convolution. Moreover, we invent patch-wise self-attention with stacked multi-heads to enrich the sensor feature representation. SensorNet is generalizable to pervasive applications with any number of sensor inputs, and is much smaller than the state-of-the-art self-attention and convolution hybrid baseline (0.83 M vs. 3.87 M parameters) with similar performance. The experimental results show that SensorNet is able to achieve state-of-the-art performance compared with the top five models on a competition activity recognition dataset (SHL’18). Moreover, pretrained SensorNet in a large inertial measurement unit (IMU) dataset can be fine-tuned to achieve the best accuracy on a much smaller IMU dataset (up to 5% improvement in WISDM) and to achieve the state-of-the-art performance on an EEG dataset (SLEEP-EDF-20), showing the strong generalizability of our approach.

## 1. Introduction

Human behavior recognition using sensors is a fundamental problem in ubiquitous computing, human–computer interactions, and ambient assisted living. With the rise of sensor-equipped mobile devices, numerous sensors now enable seamless monitoring of contextual information based on digital traces from web interactions, infrastructure, and wearable devices [1]. Modern human behavior recognition algorithms with sensor data are used in various application areas, which include but are not limited to fitness tracking, smart homes, and healthcare support. For example, human daily activities can be recognized using inertial measurement units (IMU) [2] or by detecting sleep stages through EEG signals [3]. Research in sensor-based human behavior recognition has traditionally leveraged a broad array of methodologies. While statistical machine learning algorithms such as support vector machines, decision trees, and ensemble methods have been extensively used due to their effectiveness in smaller datasets and interpretable nature, the advent of deep learning has introduced more sophisticated tools. Architectures such as Convolutional Neural Networks (CNNs) [4], Recurrent Neural Networks (RNNs) [5], and hybrid models containing both architectures [6] have set new standards, especially in handling large-scale data. Following the development of attention mechanisms, attention-based models have been widely adopted for representing sensor data.

There are several challenges in the process of training these deep neural networks across diverse pervasive applications. In this study, we focus on one specific challenge: the poor portability of pretrained neural networks on new pervasive application where sensor data are limited. We observe two main issues contributing to the challenge. (1) Varying numbers of sensor channels (**Issue 1**). For example, twenty EEG channels, two EOG channels, three EMG channels, and one ECG channel are used for measuring the electrical activity of the brain [7], while a six-axis IMU device (including a three-axis accelerometer and a three-axis gyroscope) is used to measure body movements [8]. (2) Varying sensor signal lengths (**Issue 2**). For instance, the IMU can be captured at a wide variety of frequencies, such as 10 Hz to 100 Hz, with the data lengths of 1×600 to 1×6000. **Issue 1** requires rebuilding the first network layer with a different input channel size, while **Issue 2** may have a significant impact on the design of the feature representation architecture.

Each issue can be mitigated individually by state-of-the-art models. For **Issue 1**, neural networks based on self-attention are a natural candidate solution, as the self-attention mechanism weights input sensor channels based on the pairwise importance without requiring a fixed number of neurons in the input layer (trainable parameters). This makes it flexible enough to deal with any input shape. For **Issue 2**, a CNN is straightforward to use; the kernel can simply be applied a different number of times depending on the size of the input, meaning that the output of the convolution operation scales accordingly. However, the portability challenge persists when both issues are present at the same time. Specifically, the initialization of CNN kernels is task-specific, as the kernel depth (input channel size) is bound to the specific sensor channels and as such cannot be used directly across diverse pervasive applications. Furthermore, despite the success of self-attention-based neural networks at large scales, their performance is still below similarly sized CNNs when trained on smaller amounts of data [9]. It should be noted that most of sensor datasets in pervasive applications are small because the data collection is expensive and not always efficient.

When attempting to use convolution in a way that is complementary to attention or vice versa, the requirement of modifying the input size of CNN in the earlier layer can reduce the performance of pretrained models across diverse pervasive applications [10]. In general, the performance of pretrained models are heavily influenced by local features rather than global ones [11]. As the early layers extract and represent local features, it is usually recommended to freeze the early layers during fine tuning, and only retrain the latter layers [12].

In this study, we propose SensorNet, where the local multi-scale context information extraction is based on the convolution mechanism and the kernel weights are calculated from the patch-wise relationships across the sensor channels through the self-attention approach. In particular, we define an Attention Kernel, which is used to extract sliding patches (such as sliding a kernel in a CNN) from different sensor channels. In contrast to the previous work, such as ViT with convolutional stem [13] and the Cswin transformer [14], which used early convolutions to help enhance the exploration of the intra-relationships between the patches within the same image, SensorNet learns the localized patch-wise inter- and intra-relationships across the sensor channels. It should be noted that a uniform Attention Kernel weight is assigned to the elements in the respective field of attention convolution due to the self-attention mechanism; therefore an adaptive average pooling operation is used to gain more summarized local information (i.e., intra-relationships) from each sensor channel. The major contributions of this paper are as follows:

• **Generalizable neural network.** Our work is responsible for generating a generalizable neural network for sensor feature learning across diverse pervasive applications using an adaptive attention convolution. The self-attention mechanism allows the model to fit into any number of the sensor channels input, while the convolution mechanism enables the model to represent sensor features effectively.
• **Cross-channel based patch-wise self-attention.** We invent cross-channel based patch-wise self-attention with stacked multi-heads to enrich the sensor feature representation. Specifically, the self-attention block is composed of a dense layer and an attention layer, with the former used to generate the multi-heads and the latter to learn the inter- and intra-relationships of the sensor channels using cross-channel-based patch-wise self-attention. In addition, a convolution-based multi-head approach is proposed to expand the heads while using fewer parameters than a traditional multi-head approach.
• **Extensive experiments and open-source artifacts.** Three open-source datasets are used to evaluate our proposed model. The experimental results demonstrate that our method is generalizable and practical across diverse pervasive applications.

## 2. Related Work

### 2.1. Sensor Data Representation

A sensor-based human activity recognition (HAR) might be considered as a time domain classification problem [2,15] in which different types of sensors such as Inertial Measurement Units (IMUs), health monitoring devices (e.g., heart rate sensors), and binary sensors (e.g., motion detectors, door/window sensors, switch sensors) are used to collect data [16]. On the other hand, some works have applied the Fast Fourier Transform (FFT) to short time windows in order to acquire frequency spectra of sensor data, then used them as inputs for classification algorithms [17,18]. It has been shown in recent years that sensor-based HAR using various time–frequency representations such as spectrograms can offer a rich representation of the original signal’s temporal and spectral structure, outperforming methods based on the time domain or frequency domain alone [19,20]. This has inspired us to use time–frequency spectrograms as input for sensor feature learning.

### 2.2. State-of-the-Art Backbones

Convolutional neural networks (CNNs) [21,22,23], which use convolution kernels to extract local multi-scale context information, represent a powerful technique for various tasks. Meanwhile, theoretical analysis [24] has suggested that self-attention can express the functional semantics of any convolution layer when equipped with sufficient capacity. Therefore, researchers have explored the possibility of integrating the self-attention mechanism into different tasks. There are two distinct branches of research into self-attention; one uses self-attention as building blocks in a network [25,26,27], while the other views self-attention and convolution as complementary [28,29,30].

#### 2.2.1. Models Based on Self-Attention

Models based on self-attention have been applied to a wide range of tasks, including natural language processing [31], image recognition [32], and signal classification [15]. For instance, given suffuicient data, Vision Transformer (ViT) [32] is able to treat an image as a sequence, which can be then used as an input to transformer models to achieve competitive image recognition results. Similarly, the Self-Supervised Audio Spectrogram Transformer (SSAST) uses a patch-based joint discriminative and generative framework to learn the spectrogram representation for audio and speech classification [33]. A number of studies have used self-attention as the basic building block when exploring the expressive ability of self-attention in the generation of long-range dependencies [34,35]. Many studies have suggested that self-attention could be used as a stand-alone primitive in models to replace convolutions [25,36,37].

#### 2.2.2. Attention plus Convolution

To achieve more stable training, ViT with a convolutional stem [13] has been proposed to add convolutions at the early stage. To reduce the computational complexity of self-attention, CvT [9] uses the stride convolution in the tokenization process. By using a convolutional based positional encoding technique, CSwin Transformer [14] can improve the performance of downstream tasks. Revealing the strong underlying relationship between self-attention and convolution, ACmix [38] proposes a way to integrate self-attention and convolution in parallel with learnable weights so as to maximize both benefits while achieving strong performance. ACmix, along with many other recent self-attention and convolution hybrid models, use lightweight standard convolutions to improve model performance; however, they cannot be extended to fit any number of sensor channel inputs. Inspired by ACmix, our proposed SensorNet explores the possibility of employing attention modules and utilizing cross-channel-based patch-wise relational information to enhance the functionality of convolutional networks.

## 3. Method

We propose a novel customized attention convolution to replace the standard convolution without losing model performance. The strength of a standard CNN lies in its ability to extract the local multi-scale context information (i.e., intra-channel relationships), while the self-attention method captures the pairwise interactions between channels over the sensors (i.e., inter-channel relationships). We believe that with a smaller attention kernel and an adaptive average pooling operation between and among sensor channels, the model can learn from both inter- and intra-channel relationships without adversely affecting model performance. We observe that the spectrogram is more efficient in expressing the signal than other features that express the signal only in the time or frequency domain, as it provides both benefits and shows the relationship between time, frequency, and amplitude in a direct manner [39]. In light of the progress in computer vision during the last decade (e.g., CNN and ViT), the possibility of representing sensor signals as images opens up many options. SensorNet is designed based on sensor signal spectrograms. All sensor data are converted to spectrograms, then attention and convolution based techniques are used to process the spectrograms. For convenience, we list the notation used in this work in Table 1.

### 3.1. Overall Architecture

**Figure 1:**
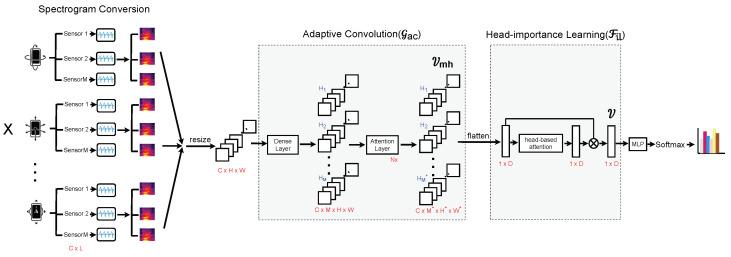
The overall architecture of SensorNet.

SensorNet (see Figure 1) consists of two major parts, namely, *adaptive convolution* $G_{ac}\left(X\right)=V_{mh}$ and *head-importance learning* $F_{il}\left(V_{mh}\right)=V$, where *X* is a sequence of sensor data vectors, $V_{mh}$ is a multi-head feature map, and V is a flattened feature vector.

Given a sequence of sensor data vectors $X=\left\{x_{1},x_{2},\cdots ,x_{C}\right\}\in R^{C\times L}$ that is converted to spectrograms $S=\left\{s_{1},s_{2},\cdots ,s_{C}\right\}\in R^{C\times H\times W}$, *S* is then mapped to $V_{mh}=\left\{\left[v_{1}^{1},v_{2}^{1},\cdots ,v_{C}^{1}\right],\left[v_{1}^{2},v_{2}^{2},\cdots ,v_{C}^{2}\right],\cdots ,\left[v_{1}^{M^{*}},v_{2}^{M^{*}},\cdots ,v_{C}^{M^{*}}\right]\right\}\in R^{C\times M^{*}\times H^{*}\times W^{*}}$ by a self-attention-based adaptive convolution module $G_{\mathrm{ac}}$ (see details in Section 3.3). This is followed by a head-importance learning module $F_{il}$, where each head generated from the self-attention is weighted based on its importance (see details in Section 3.4). After $V\in R^{1\times D}$ is obtained, classification is performed using two fully-connected layers (MLP) consisting of RELU and Softmax, with the cross-entropy used as the loss function when optimizing the classification.

### 3.2. Signal Conversion to Spectrogram Images

We convert raw signals collected from sensors into spectrogram images, a crucial step in our feature learning process. This conversion includes using the Short-Time Fourier Transform (STFT) to transform time domain signals into a time–frequency representation. The process involves signal segmentation, application of a window function, Fourier transformation, magnitude spectrum computation, and logarithmic scaling, resulting in spectrograms with the x-axis representing time and the y-axis representing frequency.

Using spectrogram images for feature learning offers several advantages:

• **Rich Representation:** Spectrograms capture both temporal and spectral information, providing a comprehensive representation.
• **Enhanced Pattern Recognition:** Spectrograms facilitate the identification of patterns and structures not apparent in raw time domain data.
• **Noise Robustness:** Transformation to the time–frequency domain enhances signal-to-noise ratio, making features more robust against noise and signal variations.
• **Compatibility with Convolutional Neural Networks (CNNs):** Spectrograms can be treated as images, making them suitable for CNNs, which are highly effective for image analysis tasks.

### 3.3. Adaptive Convolution Module

A self-attention module can be viewed as a block with two layers, namely, the dense layer and the attention layer [38]; thus, we design our self-attention-based convolution module in a similar fashion while being adaptive to the sensor channel (sensor) number.

**Figure 2:**
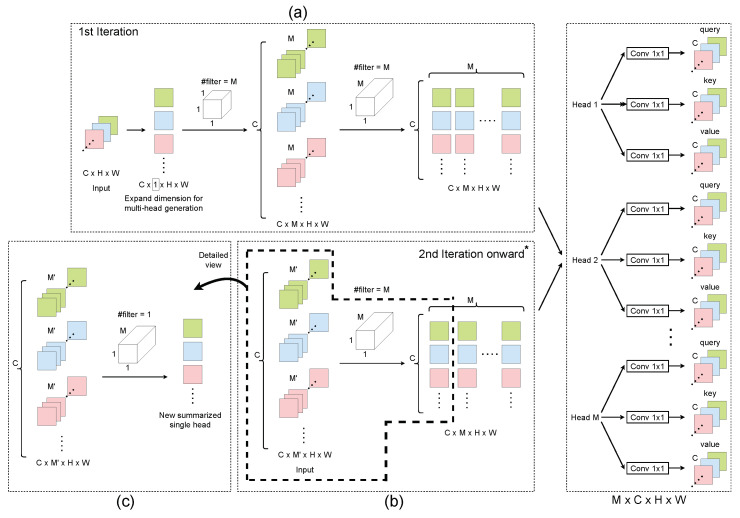
Architecture of the dense layer. * The last layer squeezes the features from the channel dimensions to obtain the global features. (**a**) shows the first iteration where the input tensor is expanded to facilitate the generation of multiple independent attention heads through a double-layer convolution block. (**b**) illustrates the procedure from the second iteration onward, where a single-layer convolutional block is used for generating the multiple attention heads, continuing the process of feature refinement. (**c**) provides a detailed view of how each attention head is further processed to summarize and refine the features extracted, helping enhance the network’s ability to focus on relevant features. The detailed introduction provided in the section on the “dense layer” effectively reflects the operations and configurations depicted in the figure.

**Dense layer.** Initially, 1×1 convolutions are conducted in the dense layer to project the input spectrograms as a query (Q), key (K), and value (V), with a multi-head method applied to stabilize self-attention learning. The adaptive convolution module is stacked many times; at the first iteration, a dummy axis is introduced to expand the input tensor of the dense layer, which results in a change of the input size from $R^{C\times H\times W}$ to $R^{C\times 1\times H\times W}$. The expanded dimension is used to generate the independent attention heads. After that, as shown in Figure 2a, a double-layer convolutional block is used to expand the individual head $R^{C\times 1\times H\times W}$ to the desired head number *M* ($R^{C\times M\times H\times W}$). After the first iteration, the multiple heads are generated through a single-layer convolutional block, as shown in Figure 2b.

*Why we need a double-layer structure at the first iteration:* For a single head representation input at the first iteration, the first convolutional layer is used to expand the individual head ($R^{C\times 1\times H\times W}$) to the desired head number *M* ($R^{C\times M\times H\times W}$) by *M* groups of 1×1×1 filter. Each channel value is related to a scalar filter value $n_{ij}$ to mitigate high correlation among the channels, where i∈M,j∈C. After that, linear combination is performed across channels in the second convolutional layer, where *M* groups of 1×1×M filters are used to produce the *M* different heads, which alleviate the correlation.

**Figure 3:**
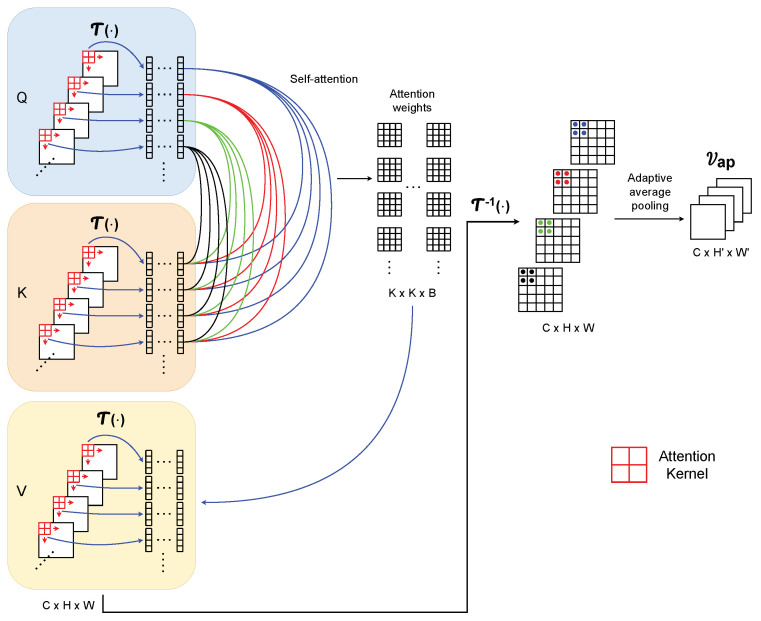
Architecture of the attention layer.

**Attention layer.** Compared with the self-attention mechanism of ViT, which learns visual representations through intra-patch interactions between the patches within the same image, our adaptive convolution module (as shown in Figure 3) learns the sensor data representations explicitly from inter-patch interactions between the patches across different spectrograms. In this work, a patch is defined as a local block extracted by sliding the *Attention Kernel* from different spectrograms (by default, we set the kernel size as 2×2), with the patch size determined as follows:

(1) Patch_Size=∏(Attention_Kernel_size).

**Figure 4:**
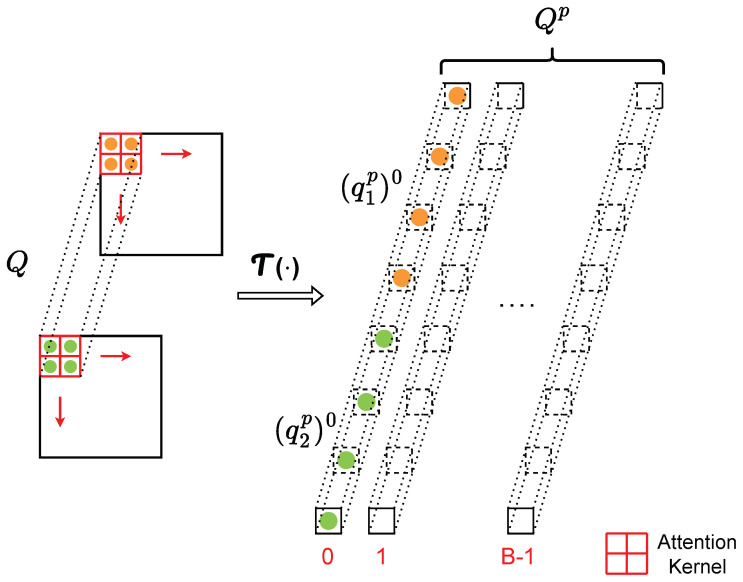
Example of the patches in Q.

All the patches in the spectrograms are eventually transformed to the shape (C,Patch_Size,B), as shown in Figure 4, with *B* the total group number of such patches in each spectrogram:

(2) $$B=\prod_{d\in 0,1}\left\lfloor \frac{Spectrogram[d]-(Attention\_Kernel[d]-1)-1}{Stride}+1\right\rfloor ,$$

where Spectrogram refers to the shape [H,W] of the input, Attention_Kernel refers to a kernel of a given size (e.g., [2,2]), and Stride represents the expected overlapping size of the sliding operation. Here, *d* is the index of the spectrogram, for example, (Spectrogram[d],d=0)=H.

To be more specific, considering a self-attention module with a single head, we first extract the respective patches from *Q*, *K*, and *V*. After assigning the Patch_Size, groups *B* of the patches can be obtained from each channel (i.e., H×W spectrogram) in *Q*, *K*, and *V*. Thus, *C* channels can extract patches with shape (C,Patch_Size,B) in each of *Q*, *K*, and *V*:

(3) $$Q^{p}=T\left(Q\right),K^{p}=T\left(K\right),V^{p}=T\left(V\right)$$

where $Q^{p},K^{p},V^{p}$ are the updated *Q*, *K*, and *V* after patch extraction and T(•) represents the patch extraction operation. Next, the patch pairwise attention score between *C* for each group is computed as follows:

(4) $$A{\left(q_{i}^{p},k_{j}^{p}\right)}^{b}=\frac{exp\left(Sigmoid\left({\left({\left(q_{i}^{p}\right)}^{b}\right)}^{T}{\left(k_{j}^{p}\right)}^{b}\right)\right)}{\sum_{f=0}^{C}exp\left(Sigmoid\left({\left({\left(q_{i}^{p}\right)}^{b}\right)}^{T}{\left(k_{f}^{p}\right)}^{b}\right)\right)}$$

where b={0,1,⋯,B−1} represents the group index and i,j∈C. This is followed by calculating the output of the self-attention:

(5) $$Attention{\left(Q^{p},K^{p},V^{p}\right)}^{b}=\sum_{i,j=0}^{C}\left(A{\left(q_{i}^{p},k_{j}^{p}\right)}^{b}{\left(v_{j}^{p}\right)}^{b}\right).$$

In order to stack the attention layers conveniently, we reverse the shape of $Attention(Q^{p},K^{p},V^{p})$ back to the shape of the layer input (i.e., (C,Patch_Size,B)→(C,H,W)):

(6) $$V^{\prime }=T^{-1}\left(Attention\left(Q^{p},K^{p},V^{p}\right)\right)$$

where $T^{-1}\left(•\right)$ represents a shape reverse operation. As a uniform *Attention Kernel* weight is assigned to the elements in the same patch due to the self-attention mechanism, an adaptive average pooling operation with an automatically selected stride and kernel size is used to gain more summarized local information:

(7) $$V_{ap}=AdaptiveAvgPool\left(V^{\prime }\right).$$

Moreover, we use multi-head attention to stabilize the self-attention learning process. In this work, the multi-head feature map is generated by

(8) $$V_{mh}^{\prime }=\overset{M}{\underset{m=1}{S}}\left({V_{ap}}^{\left(m\right)}\right),$$

where *S* represents a stack operation and m∈{0,1,⋯,M−1} is the head number.

For extraction of $V_{mh}$ (shown in Table 2), three iterations of the attention layer are taken. The first two capture the local features of the independent multiple heads from different sub-feature spaces, while the last layer squeezes the features from the channel dimensions to obtain the global features.

Discussion of multi-head attention. Standard multi-head attention [35] concatenates every single-head attention from different representation subspaces, resulting in final aggregated values using a fully-connected feed-forward layer, as shown in Equation (9):

(9) $$MultiHead\_std=Concat\left(head_{1},\cdots ,head_{M}\right)W^{f}$$

where $W^{f}$ is a fully-connected feed-forward layer and *M* is the number of heads. Our multi-head attention mechanism stacks every single head together to create a multi-channel feature map, with each channel representing an individual signal head. We then apply a convolution-based module to process the feature map, as shown in Equation (10):

(10) $$MultiHead\_ours=Stack\left(head_{1},\cdots ,head_{M}\right)⊗W^{k}$$

where ⊗ is a convolution process and $W^{k}$ is a convolution filter (as shown in Figure 2c).

In general, a convolutional layer is much more specialized and efficient than a fully connected layer. Each neuron in a fully connected layer is connected to every other neuron, and each connection has its own weight, making it quite expensive to operate in terms of memory (weights) and computation (connections). Conversely, each neuron in a convolutional layer is only connected to a few nearby neurons from the previous layer, and the weights and local connections are the same for all neurons. Due to the lower number of parameters, convolution-based multi-head attention is more efficient than the standard approach in terms of reduced model size (parameters). Based on this idea, all of the stacked multi-head values are aggregated to generate a new summarized single head using an individual 1×1×M filter (i.e., #filters=1), as shown in Figure 2c, while more heads are generated to benefit the learning process when #filters>1, as shown in Figure 2b.

### 3.4. Head-Importance Learning

Due to the fact that multiple heads do not always leverage their theoretically superior expressiveness over vanilla attention [40], we design a head-importance learning block to assign higher weights to the most important heads. The head-importance learning block $F_{il}$ is defined as

(11) $$F_{il}=\sigma (W\left(V_{mh}\right),$$

where *W* denotes a single fully connected layer and σ is a Sigmoid activation function. First, $V_{mh}\in R^{256\times 1\times 1}$ is flattened and linearly projected to a same-dimensional vector ($R^{1\times 256}$). After that, a Sigmoid function is used to map this vector between 0 and 1, and V is updated accordingly:

(12) $$V=F_{il}\times V_{mh}.$$

The head-importance function is a significant component of SensorNet, as it allows the model to dynamically assign different levels of importance to each attention head. This mechanism is crucial, as in practice not all attention heads contribute equally to the model’s performance. Certain heads may capture more relevant features than others; by identifying and emphasizing these heads, the model can enhance its focus on the most informative aspects of the data.

The head-importance function operates by applying a fully connected layer followed by a Sigmoid activation function to the multi-head feature map. This process results in a set of weights that indicate the relative importance of each head. These weights are then used to adjust the contributions of the heads during the attention calculation, effectively prioritizing the most valuable features.

By incorporating the head-importance function, SensorNet achieves better performance and efficiency. This approach ensures that the model leverages the most critical information from the attention heads, leading to improved accuracy and robustness in sensor feature learning. Additionally, this method helps to reduce the computational load by allowing the model to focus on the heads that matter most, thereby allocating resources more effectively.

## 4. Experiments

Our experiments contained two main parts: (1) Model performance evaluation, in which the performance of SensorNet was evaluated in comparison to the state-of-the-art baselines on an inertial measurement unit (IMU) dataset (Q1) and a comprehensive ablation study was conducted to obtain a better understanding of SensorNet’s behavior (Q2–Q6); and (2) Model portability evaluation, where the difference in performance when applying the pretrained SensorNet to the same type of data (IMU) and a different type of data (electroencephalography) was investigated to verify SensorNet’s portability.

### 4.1. Datasets and Implementation Details

SHL 2018 challenge. The SHL 2018 challenge [41] is an open-source dataset containing a variety of sensors used to recognize activities, including accelerometers, gyroscopes, magnetometers, linear accelerations, gravity, orientations (quaternions), and ambient pressures. This dataset contains eight activities: Car, Bus, Train, Subway, Walk, Run, Bike, and Still. The size of the dataset is 7.4 GB, and all the data were collected at a sampling rate of 100 Hz.

WISDM. The WISDM dataset [42] is an open-source dataset consisting of data collected from accelerometers and gyroscopes on a smartphone and smartwatch. This dataset contains eighteen human daily activities: walking, jogging, climbing stairs, standing, kicking, dribbling, playing catch, typing, writing, clapping, brushing teeth, folding clothes, eating pasta, eating soup, eating sandwich, eating chips, and drinking. The size of the dataset is 895 MB, and all data were collected at the sampling rate 20 Hz. In this experiment, only the three-axis accelerometer data from the smartphone were used to evaluate SensorNet’s performance on a single sensor device.

SLEEP-EDF-20. The Sleep-EDF-20 dataset [43] is an open-source dataset that contains data files for 20 subjects. The data were collected from an EEG (from Fpz-Cz and Pz-Oz electrode locations), EOG (horizontal), and submental chin EMG. This dataset consists of sleep stages W,R,1,2,3,4,M. In this context, W,R, and *M* represent the Wake stage, REM (Rapid Eye Movement) stage, and Movement stage, respectively. The remaining stages 1,2,3, and 4 correspond to specific sleep stages, as follows: Stage 1 represents the lightest stage of non-REM sleep, characterized by a relatively light sleep state; Stage 2 is characterized by the presence of sleep spindles and K-complexes; Stage 3 is marked by the presence of high-amplitude delta waves, and is also known as slow-wave sleep or deep sleep; finally, Stage 4 represents another stage of deep sleep with predominantly delta waves. The size of the dataset is 2.0 GB and all data were collected at the sampling rate of 100 Hz. To evaluate the performance of SensorNet, we adopted a subject-wise 20-fold cross-validation, following previous studies [44,45,46].

Training: We implemented SensorNet with PyTorch 1.13.1. All experiments were conducted using a single NVIDIA RTX 3090 GPU (Santa Clara, CA, USA). We used the Adam optimizer for all datasets. The training consisted of 100 epochs with a learning rate (lr) of $1\times 10^{-3}$ for training from scratch and $1\times 10^{-4}$ for transferring and fine-tuning. The random seed and batch size were set to 123 and 128, respectively.

### 4.2. Model Performance Evaluation

In the SHL 2018 Challenge dataset, **four** sensor channels are used: (1) The vector norm of the three-axis accelerometer; (2) The y-axis of the gyroscope; (3) The vector norm of the three-axis magnetometer; and (4) The w-axis of the orientation. The model input shape is (4, 6000), where 6000 is the signal length.

We evaluated the performance of our solution against the top five submissions to the SHL 2018 challenge, with the goal of understanding the following questions:

• **Q1: How does** **SensorNet** **compare to the other baseline models?**
  Table 3 shows the comparison of SensorNet with the top five algorithms. Specifically, SensorNet’s accuracy on the testing set was 91.1%, which is slightly lower than Rank 1 (93.9%) and Rank 2 (92.4%), but showed considerable improvement over Rank 3 (88.8%). It should be noted that all of the top five submission algorithms were specifically designed for the SHL 2018 challenge, while SensorNet was developed to be a generic solution. In addition, SensorNet has a model size of **3.5 MB**, which is far smaller than the model sizes of Rank 1 (**500 MB**) and Rank 2 (**43 MB**). This suggests that SensorNet is more likely to be deployable on a variety of mobile devices.
• **Q2: How does the proposed adaptive convolution perform?**
  The ACmix block [38] is a SOTA hybrid approach to integrating self-attention and convolution modules. In **Q2**, we compare SensorNet’s performance with the adaptive convolution block as well as with the ACmix block. Table 4 (Q2) shows that our proposed adaptive convolution block (91.1%) has very similar performance to the ACmix block (90.7%), demonstrating the effectiveness of our proposed adaptive convolution.
• **Q3: How does the size of the spectrogram affect** **SensorNet****’s performance?**
  Based on Table 4 (**Q3**), it can be observed that the size of the spectrogram affects SensorNet’s performance. We measured spectrograms with sizes of 16×16, 32×32, 48×48, 64×64, and 80×80, finding that model performance does not increase monotonically with increasing size of the spectrogram. Accuracy improves from 82.6% to 91.1% when the spectrogram size increases from 16×16 to 48×48; however, as the size continues to increase, the accuracy tends to flatten. This behavior can be explained by the optimal balance achieved at 48 × 48, where sufficient detail is captured without excessive noise. Beyond this size, the risk of overfitting and increased computational complexity outweigh the benefits, leading to decreased accuracy. Therefore, while larger spectrogram sizes can capture more detailed features, they introduce side effects such as higher computational demand and more potential for overfitting, highlighting the importance of selecting an optimal spectrogram size. The adaptive selection of the spectrogram size for diverse pervasive applications could be a promising research direction.
• **Q4: How does the size of the attention kernel affect model performance?**
  According to Table 4 (**Q4**), increasing the size of the attention kernel reduces the model’s performance. A model with an attention kernel of 2×2 has the best accuracy (91.1%); however, the performance decreases dramatically when using a larger kernel size such as 4×4 (86.7%) or 6×6 (83.4%). The strength of CNN lies in its ability to extract the local multi-scale context information. While the attention method captures the pairwise interactions between channels over the sensors, it misses the intra-relationships within the same kernel receptive field. While using a smaller attention kernel may not produce a significant negative impact on the model’s performance, increasing the attention kernel loses more local information and results in decreased accuracy.
• **Q5: How does head-importance learning affect model performance?**
  According to Table 4 (**Q5**), head-importance learning positively impacts model performance. Based on our evaluation of model performance with and without the head-importance learning block, we find that model performance is noticeably improved when the head-importance learning block is employed. The accuracy increases from 88.2% without using the head-importance learning block to 91.1% when using the head-importance learning block. This result confirms previous findings [40] that not all heads contribute equally to model performance. Our proposed head-importance learning block allows the most important heads to be selected adaptively.
• **Q6: How does the convolution-based multi-head attention compare to the standard one?**
  In this evaluation, we did not apply the head-importance learning block, as it is not designed for use with the standard multi-head attention. All other settings remained unchanged (i.e., using the same number of heads in both methods). As shown in Table 4 (**Q6**), our convolution-based approach has similar performance (88.2%) to the standard multi-head attention (87.7%). This result indicates that convolution-based multi-head attention is capable of achieving higher performance with lower model parameters (**0.83 M vs. 3.87 M parameters**), potentially facilitating mobile device deployment.

### 4.3. Model Portability Evaluation

#### 4.3.1. The Performance Evaluation on WISDM

The WISDM dataset uses **one** sensor with **three** channels (the x, y, and z axes of the accelerometer). The model input shape is (3, 80), where 80 is the signal length.

We investigated the performance when applying SensorNet trained on the SHL 2018 dataset to WISDM, a smaller dataset with the same type of data (IMU data).

According to Table 5, SensorNet^5^ achieves the highest accuracy (85.4%) compared to other deep learning models (CNN, BiLSTM, ConvLSTM, LSTM), demonstrating that SensorNet outperforms CNN and RNN models in terms of sensor feature representation. It should be noted that the models compared in this part were all trained from scratch.

In addition, SensorNet^5^ with the adaptive convolution block (85.4%) obtains very close accuracy to SensorNet $\mathrm{with}\;\mathrm{the}\;\mathrm{ACmix}\;{\mathrm{block}}^{1}$ (86.0%), demonstrating that the adaptive convolution block can achieve SOTA performance on a small dataset. It should be noted that the ACmix block cannot be used to adaptively fit datasets with different numbers of input channels, e.g., the first layer has to be changed and retrained.

**Figure 5:**
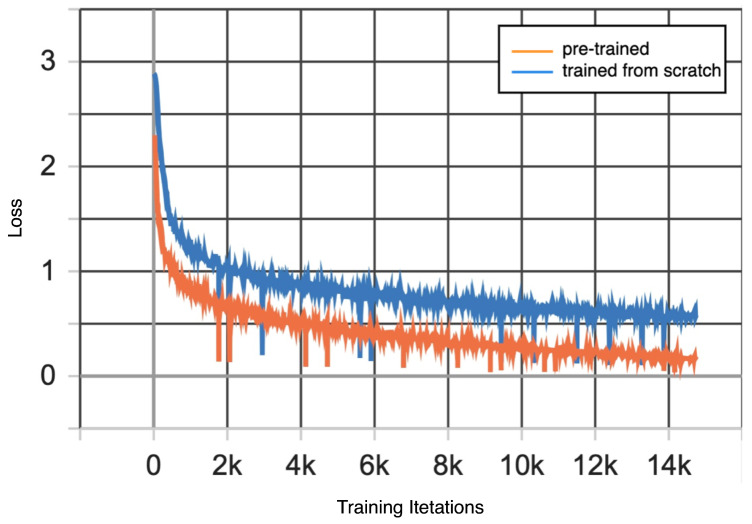
Loss comparison.

Compared with SensorNet^5^ trained from scratch (85.4%), the pretrained SensorNet^4^ with fine-tuning has better accuracy (87.1%), demonstrating its ability to transfer knowledge across similar tasks. Figure 5 illustrates that the pretrained SensorNet^4^ with fine-tuning (the orange curve) has better convergence and lower loss values compared to SensorNet^5^ trained from scratch (the blue curve).

With the input layer modified to fit WISDM, it is interesting to observe that the performance of SensorNet $\mathrm{with}\;\mathrm{ACmix}\;{\mathrm{block}}^{1}$ achieves even higher accuracy (86.0%) than SensorNet $\mathrm{with}\;\mathrm{the}\;\mathrm{ACmix}\;{\mathrm{block}}^{3}$ (82.1%), and is very close to the performance of SensorNet $\mathrm{with}\;\mathrm{the}\;\mathrm{ACmix}\;{\mathrm{block}}^{2}$ (86.3%). This observation confirms the findings of previous work [11], i.e., the performance of a pretrained model is heavily influenced by local features extracted from the earlier layers rather than by global ones extracted from the later layers. Modifying an earlier layer of a pretrained model can invalidate the model weights learned from previous tasks, as shown by the results of SensorNet $\mathrm{with}\;\mathrm{the}\;\mathrm{ACmix}\;{\mathrm{block}}^{3}$, making the fine-tuning process with a large learning rate similar to training a new model from scratch, as shown by the results of SensorNet with the ACmix block^2^. This experiment further demonstrates the superiority of the pretrained SensorNet in terms of its ability to transfer to a small sensor dataset regardless of input size.

#### 4.3.2. The Performance Evaluation on SLEEP-EDF-20

The SLEEP-EDF-20 dataset uses **two** sensors with **three** channels: EEG (Fpz-Cz), EEG (Pz-Oz), and EOG (horizontal). The model input shape is (3, 3000), where 3000 is the signal length.

We further investigated the performance when applying the pretrained SensorNet obtained from SHL 2018 (**pretrained** **SensorNet** **(SHL 2018)**) to SLEEP-EDF-20, a smaller dataset with very different data types (EEG and EOG signals). According to Table 6, the pre-trained SensorNet (SHL 2018) (85.9%) is capable of achieving SOTA performance (86.4%), again showing the portability of the model. Additionally, the pretrained SensorNet (SHL 2018) (85.9%) achieves slightly higher accuracy than SensorNet trained from scratch (85.0%). On the one hand, this result suggests that the pretrained SensorNet obtained from the IMU data (SHL 2018) does not seem able to provide very much related knowledge on the feature representation learning for the EEG data (SLEEP-EDF-20). The slight improvement, however, indicates that the self-attention mechanism in SensorNet may learn some valuable relationships between the different sensor channels regardless of the type of data (we call this data-agnostic learning). These results raise another interesting concern regarding how a pretrained SensorNet obtained from SLEEP-EDF-20 (EEG and EOG) would perform on the WISDM dataset (IMU).

#### 4.3.3. Extended Evaluation of the Pretrained SensorNet Obtained from SLEEP-EDF-20

**Figure 6:**
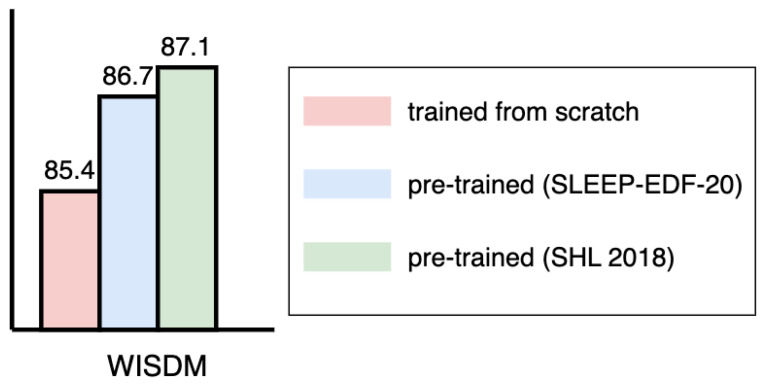
Performance of the pretrained SensorNet obtained from SLEEP-EDF-20.

Figure 6 presents the model performance on WISDM using the pretrained SensorNet obtained from SLEEP-EDF-20 (**pretrained** **SensorNet** **(SLEEP-EDF-20)**). The performance when using the pretrained SensorNet (SLEEP-EDF-20) (86.7%) is lower than when using the pretrained SensorNet (SHL 2018) (87.1%), indicating that the pretrained SensorNet with the same type of data can learn more related knowledge.

Moreover, we further confirmed SensorNet’s data-agnostic learning capability. A higher accuracy (86.7%) is achieved when applying the pretrained SensorNet (SLEEP-EDF-20) to WISDM (85.4%) than when using SensorNet trained from scratch. According to this result, even though the pretrained SensorNet on EEG and EOG data (SLEEP-EDF-20) cannot gain very much useful related knowledge on the feature representation from the IMU data (WISDM), the self-attention based convolution in SensorNet can still learn implicit patch-wise relationships across various sensor channels, thereby improving model performance. Investigating a systematic way of quantifying and exploring such data-agnostic learning abilities could be another promising research direction.

### 4.4. Discussion

#### 4.4.1. Adaptability to Sensor-Based Pervasive Applications

The experimental results from the SHL 2018 (four IMU sensors), WISDM (one IMU sensor), and SLEEP-DEF-20 (two bioelectric sensors) datasets demonstrate that SensorNet can handle diverse sensor-based pervasive applications without having to modify the model structure except for the last classification layer. In particular, the proposed adaptive convolution block that integrates self-attention and convolution proves to be capable of achieving SOTA feature representation learning performance.

#### 4.4.2. Impact of Different Sensor Data Dimensions

The experimental results from SHL 2018 (6000 datapoints per channel), WISDM (80 datapoints per channel) and SLEEP-DEF-20 (3000 datapoints per channel) demonstrate that the performance of SensorNet is not sensitive to the input data dimensions. In particular, while the adaptive convolutions learn the inter- and intra-relationships among different sensor channels via the self-attention mechanism, the small size of the attention kernel (such as a 2×2 attention kernel) can adapt to spectrograms from different sensor data dimensions.

#### 4.4.3. Summary of Results and Flexibility of SensorNet

This study has presented SensorNet, an adaptive attention convolutional neural network designed for sensor feature learning across diverse pervasive applications. The experimental results demonstrated that SensorNet achieves state-of-the-art performance while maintaining a compact model size. The key findings are summarized below:

1. Performance Across Datasets: SensorNet achieves competitive accuracy on the SHL 2018 challenge dataset, outperforming several baseline models and showing strong generalizability. This is further evidenced by its high performance on the WISDM and SLEEP-EDF-20 datasets, demonstrating its adaptability to different sensor types and data characteristics.
2. Generalizability: The model’s architecture, integrating self-attention with convolutional mechanisms, allows it to handle varying numbers of sensor channels and diverse sensor signal lengths. This flexibility is crucial for applications with heterogeneous sensor data, making SensorNet a versatile solution for real-world deployment.
3. Efficient Model Size: Despite its high performance, SensorNet maintains a significantly smaller model size compared to other state-of-the-art models. This efficiency is achieved through the use of adaptive attention convolutions, which reduces the number of parameters while preserving accuracy. This compactness facilitates deployment on resource-constrained devices such as mobile and wearable technology.
4. Handling Diverse Sensors: SensorNet’s ability to handle diverse sensors stems from its innovative architecture. The adaptive convolution module and multi-head attention mechanism enable the model to effectively learn both inter- and intra-sensor relationships. This capability is critical for integrating and processing data from various sensor types, enhancing the model’s robustness and applicability.

In conclusion, SensorNet provides a robust, efficient, and generalizable framework for sensor-based human behavior recognition, addressing key challenges in the field and paving the way for future research and applications in pervasive computing and beyond.

## 5. Conclusions

In this paper, we present SensorNet, a generalizable framework for learning sensor feature representation that can be adapted to various sensor-based pervasive applications. In particular, SensorNet’s novel integration of self-attention and convolution modules allows the model to fit into different sensor input sizes and effectively represent sensor features. Our experimental results demonstrate that SensorNet achieves the state-of-the-art performance and is more efficient than the baseline multi-head solution while having better accuracy. In addition, SensorNet has good portability to other pervasive applications. We argue that such improvements are essential for recognizing human behavior through sensor-based methods, and are appealing for future explorations of opportunities leveraging SensorNet for a variety of sensor-based applications. For example, it may be of interest to investigate how SensorNet can be applied to improve perception, prediction, and planning models in automated driving using multi-sensor fusion involving Lidar, radar, and cameras.

## Figures and Tables

**Table 1:** Notation.

Symbol	Definition
L(L∈N,L>1)	Length of the sequence of sensor data
C(C∈N,C>1)	Number of channels
H(H∈N,H>1)	Height of the input spectrogram
W(W∈N,W>1)	Width of the input spectrogram
M(M∈N,M>1)	Number of attention heads before the attention layer
$H^{*}\;\left(H^{*}\in N,H^{*}>1\right)$	Height of the adaptive convolution feature map
$W^{*}\;\left(W^{*}\in N,W^{*}>1\right)$	Width of the adaptive convolution feature map
$M^{*}\;\left(M^{*}\in N,M^{*}>1\right)$	Number of attention heads after the attention layer
D(D∈N,D>1)	Dimension of the feature vector

**Table 2:** Settings of the attention layers.

Layer	Input Size	Heads	AdaptiveAvgPool	Output Size
${\mathrm{Iteration}}^{1\mathrm{st}}$	C×1×48×48	64	(64,24,24)	C×64×24×24
${\mathrm{Iteration}}^{2\mathrm{nd}}$	C×64×24×24	128	(128,12,12)	C×128×12×12
${\mathrm{Iteration}}^{3\mathrm{rd}}$	C×128×12×12	256	$(1,1,1{)}^{1}$	256×1×1×1

^1^ Permute the AdaptiveAvgPool input size: C×256×12×12→256×C×12×12.

**Table 3:** Summary of the top five submissions to SHL 2018 and our method.

Rank	Team	Classifier	Input	Performance		Model Size (MB)
				Train	Test	
1	JSI-Deep [20]	DNN + ML	Spectrogram + features	96.0%	93.9%	500
2	JSI-Classic [47]	XGBoost	Features	93.7%	92.4%	43
−	SensorNet (Ours)	SensorNet	Spectrogram	**96.3%**	**91.1%**	3.5
3	Tesaguri [19]	CNN	Spectrogram	93.0%	88.8%	3
4	S304 [48]	MLP	Features	85.7%	87.5%	0.035
5	Confusion Matrix [49]	Random Forest	Features	96.8%	87.5%	1122

**Table 4:** Ablation study.

Questions	Methods	Accuracy
Q2	SensorNet with adaptive convolution block	**91.1**
	SensorNet with ACmix block [38]	90.7
Q3	SensorNet (16×16)	82.6
	SensorNet (32×32)	86.1
	SensorNet (48×48)	**91.1**
	SensorNet (64×64)	89.0
	SensorNet (80×80)	88.5
Q4	SensorNet with attention kernel 2×2	**91.1**
	SensorNet with attention kernel 4×4	86.7
	SensorNet with attention kernel 6×6	83.4
Q5	SensorNet with head-importance learning	**91.1**
	SensorNet without head-importance learning	88.2
Q6	SensorNet with standard multi-head	87.7
	SensorNet with convolution based multi-head	**88.2**

**Table 5:** Performance on the WISDM dataset.

Dataset	Methods	Accuracy
WISDM	CNN	84.9
	BiLSTM	84.8
	ConvLSTM	84.3
	LSTM	82.5
	SensorNet with ACmix block ^1^ [38]	86.0
	SensorNet with ACmix block ^2^ [38]	86.3
	SensorNet with ACmix block ^3^ [38]	82.1
	SensorNet ^4^	**87.1**
	SensorNet ^5^	85.4

^1^ model trained starting from scratch. ^2^ pretrained model with fine-tuning (lr: $1\times 10^{-3}$). ^3^ pretrained model with fine-tuning (lr: $1\times 10^{-4}$). ^4^ pretrained model with fine-tuning. ^5^ model trained starting from scratch.

**Table 6:** Performance comparison between state-of-the-art approaches on the SLEEP-EDF-20 dataset.

Dataset	Year	Methods	Accuracy
SLEEP-EDF-20	2021	XSleepnet2 [44]	86.4
	2020	SimpleSleepNet [45]	85.4
	2020	XSleepnet1 [44]	85.2
	2020	DeepSleepNet+ [46]	84.6
	2022	SensorNet ^1^ (ours)	**85.9**
	2022	SensorNet ^2^ (ours)	85.0

^1^ pretrained model with fine-tuning. ^2^ model trained starting from scratch.

## Data Availability

The data presented in this study are available on request from the corresponding author.
